# Epidemiological and Clinical Observations of Gonococcal Infections in Women and Prevention Strategies

**DOI:** 10.3390/vaccines9040327

**Published:** 2021-04-01

**Authors:** Ana Paula R. Costa-Lourenço, Xiaohong Su, Wenjing Le, Zhaoyan Yang, Gregory J. Patts, Paola Massari, Caroline A. Genco

**Affiliations:** 1 Department of Immunology, Tufts University School of Medicine, 136 Harrison Avenue, Boston, MA 02111, USA; ana.lourenco@tufts.edu (A.P.R.C.-L.); paola.massari@tufts.edu (P.M.); 2 Institute of Dermatology, Chinese Academy of Medical Sciences and Peking Union Medical College, 12 Jiangwangmiao Street, Nanjing 210042, China; suxh@ncstdlc.org (X.S.); lewj@ncstdlc.org (W.L.); 3 Biostatistics and Epidemiology Data Analytics Center, Boston University School of Public Health, 85 East Newton Street, Boston, MA 02118, USA; zy@bu.edu (Z.Y.); gpatts@bu.edu (G.J.P.)

**Keywords:** *Neisseria gonorrhoeae*, sexually transmitted diseases, vaccines, clinical manifestations

## Abstract

*Neisseria gonorrhoeae* is rapidly developing antimicrobial resistance. There is an urgent need for an effective gonococcal vaccine. In this study we examined epidemiological and clinical factors associated with gonorrhea in a cohort of women exposed to men with gonococcal urethritis attending the National Center for STD Control clinic in Nanjing, China, to understand the natural history and the risk factors for gonorrhea in this vulnerable population. This analysis will help identify the best target populations for vaccination, which is essential information for the development of vaccine strategies. We observed that 75% of the women in our cohort yielded a *N. gonorrhoeae* positive culture (infected women) and reported multiple sexual exposures to their infected partner. Infected women were younger than exposed but uninfected women. Contrary to the general belief that gonorrhea is asymptomatic in most women, 68% of the infected women acknowledged symptoms during their STD clinic visit, and overt inflammatory responses were detected upon medical examination in 88% of subjects. Other sexually transmitted infections were detected in 85% of subjects. This study confirmed that *N. gonorrhoeae* infections are underdiagnosed in women and, consequentially, untreated. Thus, our analysis reinforces the need to establish strategies for gonococcal prevention through the determination of the target population for a gonococcal vaccine.

## 1. Introduction

*Neisseria gonorrhoeae* is the causative agent of gonorrhea, a sexually transmitted disease (STD) with high incidence worldwide (87 million cases in 2016) [1]. Men with gonorrhea usually develop symptoms and generally seek treatment. In contrast, women who develop gonococcal cervicitis rarely report overt inflammatory symptoms [2,3]. Thus, gonorrhea in women is largely undiagnosed, under-reported, and untreated. Prolonged infections can progress into pelvic inflammatory disease (PID) with long-term sequelae (chronic pelvic/abdominal pain, tissue scarring, ectopic pregnancy, and infertility) or disseminated gonococcal infection (DGI) [2]. Other major implications of *N. gonorrhoeae* infection include exacerbated risk of transmission and increased susceptibility to other sexually transmitted infections (STIs) such as *Chlamydia trachomatis* or human immunodeficiency virus (HIV) [2]. 

Gonorrhea is effectively treated with antibiotics, but a rapid onset of antimicrobial resistance is being reported worldwide, limiting the available antibiotic choices [4]. A gonococcal vaccine has historically lagged due to several roadblocks, including lack of known correlates of protection in humans, limitations of available animal models of gonococcal genital infection, challenges in identification of protective antigens and a poor understanding of natural mucosal infection, mainly in asymptomatic women [5]. To date, a few gonococcal vaccines have been explored in humans but have shown limited efficacy [5,6,7,8,9,10]. 

*N. gonorrhoeae* human challenge studies have been restricted to male volunteers due to the high risks of complications for women upon *N. gonorrhoeae* infection. However, these studies only provide information regarding the acute phase of infection and do not address long-term immunity (the volunteers were treated at the first appearance of symptoms or signs of infection [11]). 

Studies of natural gonococcal infection in humans are a necessary component to understand how to reduce and/or prevent infection and transmission but, unfortunately, such studies in women are scarce. The current study was designed to enroll women exposed to men with gonococcal urethritis but who would not otherwise seek medical assistance for an STI based on their own judgment of symptoms or signs of such an infection. The women in our cohort are partners of men with gonococcal urethritis that visited the National Center for STD Control (NCSTD) clinic in Nanjing (China). We examined the clinical and epidemiological parameters associated with gonococcal infection in these women to better define disease manifestations in this vulnerable population. Our results demonstrate that the majority of exposed women were infected with *N. gonorrhoeae*, often presented with other STIs, and despite not presenting sufficient symptoms of an STI to voluntarily seek medical assistance, they showed signs and symptoms of overt inflammatory responses upon clinical examination. Analysis of the characteristics of this population has provided important clinical insights into the characteristics of disease presentation in women who were exposed to men with gonorrhea infection. Furthermore, these parameters of disease presentation have important implications for detection and prevention strategies in this vulnerable population. 

## 2. Materials and Methods

### 2.1. Study Design 

This cross-sectional survey study was designed with a focus on recruitment of men attending the National Center for STD Control (NCSTD) clinic in Nanjing (China) with gonococcal urethritis, and their female partners. Limiting criteria for enrollment of women included a self-reported monogamous status and absence of otherwise reportable symptoms that would have required clinic visit not prompted by the man’s diagnosis of gonorrhea. 

### 2.2. Eligibility and Enrollment

Men who reported urethral discharge for at least two days prior to the visit, who agreed to inform their partners about infection status and to invite them to participate in the study, were considered eligible for enrollment. Men received written information on ways for their partner to contact the NCSTD research team. One of the eligibility criteria for the women was to self-identify as monogamous in a one-month period prior to the man’s visit to the NCSTD. Women who were considered eligible for the study were asked to enroll as corresponding partners of the infected men. 

#### 2.2.1. Exclusion Criteria 

Subjects below 18 years of age, self-reported HIV positive, or non-Chinese speaking were excluded, as were men who only had sex with men. Women who were pregnant, residing outside of Nanjing, or unwilling to receive treatment upon clinical exam, who used azithromycin (2 weeks) or other antibiotic (4 days) before exposure, or who previously participated in this study were also excluded. 

#### 2.2.2. Inclusion Criteria

Men: intercourse with at least one female partner in the past 30 days and diagnosis of gonorrhea at clinic visit. Women: at least one episode of unprotected vaginal intercourse with the index male during a one-month time period prior to his visit to the NCSTD clinic.

### 2.3. Specimens Collected

At the time of the subject’s visit to the NCSTD clinic, first-voided urine specimens were collected. In addition, two urethral or four cervical exudate specimens were collected with cotton swabs from male or female subjects, respectively. 

### 2.4. N. gonorrhoeae Infection Diagnosis

One of the urethral or cervical exudate specimens as mentioned above was used for Gram staining to identify polymorphonuclear cells (PMNs) containing Gram-negative intracellular diplococci (GNID) and for plating on Thayer-Martin plates (Zhuhai DL Biotech, Zhuhai, Guangdong, China). Plates were incubated in candle jars at 36 °C for 24–48 h and *N. gonorrhoeae* was identified by colonial morphology, Gram stain and oxidase testing (Sangon Biotech, Shanghai, China). 

### 2.5. Routine Diagnosis of Other STIs

*Mycoplasma genitalium* and *Trichomonas vaginalis* were identified by PCR of urine specimens from men and women [12]; *C. trachomatis* was identified in urethral and cervical exudates by real-time PCR (DAAN Gene Co. Ltd., Guangzhou, China); *Ureaplasma urealyticum* was identified by culture and using the Mycoplasma IST2 (BioMerieux) kit; *T. vaginalis* and *Candida albicans* were identified by vaginal wet smear. Trichomoniasis was diagnosed by observation of motile pear-shaped cells with flagella by microscopy, and candidiasis by presence of yeast cells with pseudo hyphae. Bacterial vaginosis (BV) was indicated by clue cells detected by Gram stain [13]. *Treponema pallidum* infection was reported by Rapid Plasma Reagin (RPR) and Treponema Pallidum Particle Agglutination (TPPA) tests, and syphilis was diagnosed based on dark field microscopy (presence of lesions). Herpes Simplex Virus (HSV) infection was detected by serology using ELISA kits (HSV-1 IgM and IgG, HSV-2 IgM and IgG) (KangRun Biotechnology), and by PCR (DAAN Gene Co. Ltd.). Human Papilloma Virus (HPV) infection was identified by PCR (DAAN Gene Co. Ltd.).

### 2.6. Data Collection

#### 2.6.1. Questionnaire

Upon enrollment, all subjects were assisted by a health care professional from the NCSTD clinic in responding to a questionnaire designed to obtain information about age, marital status, race and education level. Women were also asked to provide information about the last menstrual period date, contraception methods used in the past six months, dates of exposure(s) and body site exposure to the index man in a 2-week time period prior to and after his self-reported onset of symptoms. In addition, information about other past or current diagnosed STIs was requested, including *N. gonorrhoeae*, *C. trachomatis, T. vaginalis*, *Treponema pallidum*, *C. albicans*, human papillomavirus (HPV) and BV, symptoms or manifestations of STIs (genital warts and PID). When available, information collected in the questionnaire was confirmed from the subject’s medical records. Women were also asked to report whether they observed symptoms, and if so, describe them; for example vaginal discharge, vaginal bleeding other than menses, abdominal pain, dysuria, painful intercourse, rectal pain, sore throat, fever, rash and joint pain. They were also asked to indicate the time frame of onset of any of these symptoms. 

#### 2.6.2. Clinical Parameters Examined at Enrollment 

At the time of visit, all the men presented with urethral discharge. Gonococcal urethral exudate was evaluated for the presence of PMNs by microscopy and the number of PMNs was reported per high-power field (PMN/hpf). The enrolled women underwent a routine medical examination, which evaluated vaginal and cervical discharge, cervical inflammation, pain on lateral motion of the cervix, adnexal, abdominal, hepatic and rectal tenderness, presence of inguinal nodes, genital lesions and number of PMNs in the cervical discharge as above. 

### 2.7. Statistical Analyses

Descriptive statistical analyses were used to identify associations between demographic, socioeconomic, epidemiologic data collected from the questionnaire, self-reported signs and symptoms and clinical observation reported at the clinic after medical examination with gonococcal infection by reporting median and interquartile ranges (IQR) (for age), the percentages relative to the total subject number (for the other categories), and the prevalence of *N. gonorrhoeae* culture-positive women for each observed characteristic. We performed univariate analysis by two-tailed Fisher exact test, determined *p*-value of 0.05 as significance threshold (EPI INFO), and reported the odds ratio with confidence interval, and the *p*-value [14]. Due to the limited size of this cohort, the *p*-value was >0.05 for some analyses and did not indicate a relationship among compared data.

## 3. Results

### 3.1. Study Cohort

**Figure 1:**
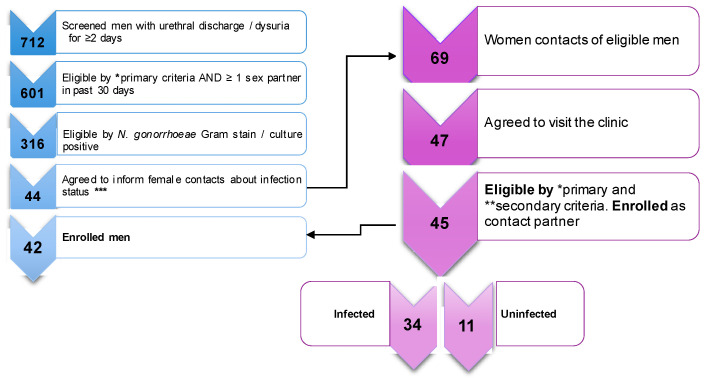
*Neisseria gonorrhoeae* cross sectional cohort enrollment scheme. Workflow describing number of screened, eligible, enrolled subjects, and *N. gonorrhoeae* infection status in enrolled women. Detailed primary and secondary criteria are also included.

In the period from December 2017 to November 2019, 601 men with symptoms of gonorrhea visited the NCSTD clinic, where they were confirmed to be infected by *N. gonorrhoeae*. Over 50% of these men were eligible to participate in the study but only a minority of subjects agreed to inform their female partners about the gonorrhea diagnosis, thus reducing the pool of potentially eligible women. Among the women who indeed contacted the NCSTD clinic, only 68% visited the clinic. Ultimately, 42 men and 45 women were enrolled (3 men enrolled 2 female partners each) (Figure 1). 

### 3.2. Assessment of Gonococcal Infection

**Figure 2:**
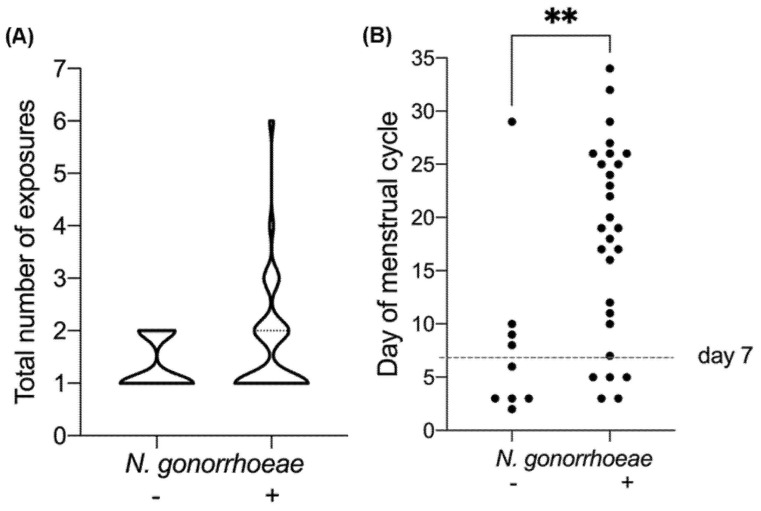
*Neisseria gonorrhoeae* infection in women exposed to men with gonococcal urethritis. Impact of (**A**) number of exposures and (**B**) menstrual cycle in *N. gonorrhoeae* culture-negative women (*N. gonorrhoeae* −) and culture positive (*N. gonorrhoeae* +) women. Infection was associated with a high number of total exposures (>2 and up to 6) (**A**). Uninfected women were mostly exposed during the follicular phase (1–11 days). The most recent exposure for each woman is indicated in (**B**). ** *p*-value 0.0065.

The cervical exudates from the 45 enrolled women were tested for *N. gonorrhoeae* by bacterial culture revealing 34 culture-positive subjects (considered infected) and 11 culture-negative subjects (considered uninfected) (Figure 1 and Figure 2A). The study design was aimed at capturing female contacts of infected men blinded in respect to their infection status prior to enrollment and thus, the culture-negative subjects could only be discerned a posteriori.

### 3.3. Epidemiology Observations from the Questionnaire

#### 3.3.1. Demographics

The overall age of the enrolled subjects, including men and women, was between 18 and 61 years; the average age of the infected women was 33 years and 39 years for the uninfected women. Over 70% of the subjects self-reported as married; other marital statuses included single and divorced. Single status was only reported by males (19%) and infected women (24% of total women and 32% of infected women). The majority of the population identified as Han, with only one woman self-identifying as a Man Zu. The education status was predominated by grade school (primary school, junior/senior high school and vocational school) (89% for women and 74% for men), and high education level (which included undergraduate and/or graduate degrees) was generally lower for women (11%) than for men (26%) (Table 1). None of the uninfected women had completed high education level.

#### 3.3.2. Chronology of *N. gonorrhoeae* Exposure

The time interval between onset of symptoms and clinic visit is referred to as time to presentation (TTP) [15]. For men, the average TTP was 7 days and for women, the time between the last exposure and the clinic visit was 9.5 days (not shown). We observed that exposed women who were infected with *N. gonorrhoeae* had more exposures than the women who remained uninfected (Figure 2A, Table 2). Over 75% of infected women had been exposed before men acknowledged onset of symptoms (Table 2).

#### 3.3.3. Physiological Parameters Relative to Exposure

Thirty-nine women indicated the last menstrual period date in the questionnaire. Among them, one was already in her menopause period and another one stated that her last menstrual period occurred 71 days before exposure to the infected man. A timeline of the women’s exposure to the infected men relative to the menstrual cycle phase [16] suggested that the majority of the uninfected women were exposed in the follicular phase, while exposure in any other cycle phase (ovulation or luteal phase) more frequently resulted in infection (Figure 2B). 

##### Contraception Methods Relative to Exposure

Analysis of contraception methods used in the six months prior to enrollment as reported in the questionnaire indicated that no contraception was used by 17 (38%) of enrolled women; 15 (33%) reported IUD use, 8 (18%) condom use, and 4 (9%) hormone-based methods. Table 3 stratifies contraception methods used by *N. gonorrhoeae* infection status. All women reported vaginal exposure (only three infected women also reported oral exposure).

#### 3.3.4. Self-Reported Signs and Symptoms 

Although women enrolled in the study did not seek treatment based on presence of symptoms, approximately 50% acknowledged vaginal discharge when prompted by the questionnaire. A range of additional symptoms such as abdominal pain, dysuria, and pain during intercourse were mentioned and were more frequently acknowledged by infected women (positive culture) (Table 4). However, 32% of infected women did not acknowledge signs and symptoms, along with 45% of uninfected subjects.

#### 3.3.5. History of STIs 

Eleven out of the 45 enrolled women reported previously diagnosed STIs other than gonorrhea (nine from the infected group and two from the uninfected group), which included BV, vaginovulvar candidiasis, and trichomonas vaginitis, with one also reporting PID (not shown). 

### 3.4. Clinical Evaluation at NCSTD

#### 3.4.1. Clinical Signs 

Upon medical evaluation, 89% of enrolled women presented overt inflammatory responses (30 infected women and 10 uninfected women), defined as vaginal discharge (87%), cervical discharge (80%) and cervical inflammation (69%) (Table 4). Vaginal and cervical discharge were classified by type (purulent and/or bloody, frequently observed in women with gonorrhea); however, discharge not associated with gonococcal infection (clear-mucoid or grey-white) was also observed in infected women (Table 4). We also observed presence of immune cells (PMNs) in the cervical discharge, which varied from high to moderate to low (≥10, 5–9, and 1–4, respectively) in both groups (Table 4). The majority of women did not present other relevant signs such as pain on lateral motion of cervix, uterine and rectal tenderness (Table S1). Of note, only one uninfected woman was free of overt inflammatory response and not all infected women presented all the signs concurrently or with the same intensity (Table 4 and Supplementary Materials Table S1).

#### 3.4.2. Diagnosed Co-Infections 

As part of routine screening at the NCSTD clinic, the enrolled women were tested for additional STIs, such as *C. trachomatis*, *M. genitalium*, *T. vaginalis*, *T. pallidum*, HSV, and HPV, as well as *U. urealyticum* and *C. albicans*, and for BV. Overall, 80% of subjects were diagnosed with concurrent multiple STIs or genital infection with a higher prevalence (>50%) in *N. gonorrhoeae*-infected women; 85% of infected women presented with up to three additional infections, while 64% of uninfected women were diagnosed with one, two, or even three other concurrent infections (namely, *C. trachomatis*, *U. urealyticum*, and *M. genitalium*). The presence of clue cells indicative of BV was also detected in 38% of infected women and 55% of uninfected women (Table 5). No evidence of *T. pallidum*, HSV and HPV infection was observed.

Although the current analysis focused on the enrolled, exposed women, availability of equivalent data for the enrolled men warrants a similar analysis of an inflammatory response and co-infections. All the enrolled men presented urethral discharge and most of them presented high levels of PMNs. Other STIs diagnosed in the men included *C. trachomatis* (12%), *M. genitalium* (10%), and *T. vaginalis* (2%); 31 men were only infected with *N. gonorrhoeae*.

## 4. Discussion

The current study was designed to examine clinical and epidemiological parameters associated with gonococcal infection in our cohort of women exposed to men with gonococcal urethritis to gain a better understanding of the natural history of gonorrhea in this vulnerable population and the risk factors contributing to this STI. This is a population that is seldom analyzed as the majority of previous studies have primarily examined women with a recognized gonorrhea infection (or who have knowledge of potential exposure). These results can, in turn, provide insights for therapeutic and preventive strategies for control of gonorrhea. For example, should vaccination (once a vaccine is developed) target sexually-active adults or would it be more beneficial if offered earlier, before young people become sexually active? Could vaccination strategies be more successful when coupled with better education about STIs during secondary school years? Is infection dependent on when a subject is exposed or the number of exposures? 

We reported that about three quarters of the women contracted gonorrhea, similar to previous studies [17,18,19]. Our demographic analysis showed that the average age of the infected women was lower than that of the subjects who remained uninfected (about 33 and 39 years of age, respectively). Two studies conducted in China, one in Beijing with symptomatic outpatients from a tertiary care hospital and the other with symptomatic individuals attending clinics in Shenzhen, have also shown that Chinese women in their thirties or older were at higher risk for *N. gonorrhoeae* infection [20]. The USA and the European Centers for Disease Control and Prevention reported a slightly lower age cut-off for risk of infection (15–25 years old) [21,22]. Infected women had a higher education level than women who remained uninfected, but for the most part, both groups only completed high school. 

When we examined infection in the context of exposure relative to the menstrual cycle, we observed a noticeable difference in the phase of the menstrual cycle between exposed women who were infected and exposed women who were uninfected. This is in agreement with previous studies [16,23,24], which have shown that changes in the female body influenced by the variation in hormonal level during the menstrual period may favor the occurrence of STIs, including *N. gonorrhoeae* [25]. Use of oral hormone-based contraceptives were only reported by infected women as opposed to other types of birth control methods, including IUDs. Since no details were available regarding the IUD type (hormone-based or copper), the relative numbers in the hormone contraceptive category may vary in our study. The role of external hormones introduced via hormonal contraceptives on susceptibility to gonococcal infection remains unclear and warrants further investigations [26,27].

As obvious as it may seem, we observed that a positive gonococcal culture result was more frequent in women who were exposed multiple times to the infected man. Indeed, 79% of the infected women reported multiple exposures; however, within the group of women who were only exposed once, 73% were infected. The same trend was observed by Platt and colleagues in a similarly designed cohort [17], in which an individual event was sufficient for the transmission of the infection, but multiple exposures were more likely to result in infection.

Additional aspects that were examined in our cohort included symptoms and other STIs. While gonorrhea is generally referred to as asymptomatic in women, our findings demonstrated that more than 50% of infected women indeed presented symptoms and signs associated with gonorrhea. This reinforces that gonorrhea presentation in women is more subtle as compared to men, and symptoms may be mild or easily misinterpreted [28]. Discharge in *N. gonorrhoeae* infections is related to the influx of PMNs to the site of inflammation [29], as indicated by a high level of PMNs detected in infected women. Upon clinical evaluation, vaginal and cervical discharge were confirmed in both infected and uninfected women, but cervical inflammation was observed in a small percent of cases. One of the main symptoms that typify gonorrhea in men, painful urination, was reported by less than 20% of infected women. The majority of women in our study presented additional STIs and it is possible that the observed symptoms were attributable to both *N. gonorrhoeae* and concurrent other STIs. For example, it is known that *C. trachomatis*, *T. vaginalis* and *M. genitalium* can induce a recognizable symptomatic response [30]. These organisms were detected in both *N. gonorrhoeae*-infected and uninfected women at variable rates and could explain symptoms in part. Another confounding factor may be a 2-fold-higher incidence of *M. genitalium* infection in the *N. gonorrhoeae*-infected women. This organism is often associated with BV [31], a multi-organism STI that can increase the risks of contracting other STIs and vice versa [32]. *U. urealyticum* infection has severe consequences during pregnancy [33], but it is not considered a true STI [31,34]. We observed that *N. gonorrhoeae*-infected women had a 2-fold-higher incidence of *U. urealyticum* than uninfected women, but it remains unclear whether this represents a risk factor for *N. gonorrhoeae* infection. 

Our demographic analysis indicated that risk of infection is not limited only to young adults with multiple and occasional sexual partners, and minorities with socioeconomic disadvantages, as shown in other cohorts [35,36,37]—a consideration which should be taken into account when planning population vaccination strategies. Despite the small size of our study compared to other studies [16,17,18,24], it presents a unique opportunity to analyze unique aspects of gonorrhea in exposed “asymptomatic” women, a population that is understudied because these individuals do not seek treatment. While some infected women may spontaneously clear the infection over time, by remaining undiagnosed and untreated, many could develop severe disease (PID) and associated consequences such as infertility or ectopic pregnancies [38,39,40,41]. Therefore, along with a continuing focus on developing vaccination strategies, it is crucial to evaluate other preventative aspects to reduce the spread of *N. gonorrhoeae* and other STIs. Our studies provide critical new clinical characteristics of gonorrhea disease in women and will guide new diagnostic and preventative strategies. 

## Figures and Tables

**Table 1:** Demographics.

Categories	Men	Women
	*n* = 42	*n* = 45 ^a^
Age	38 (31–50) ^b^	32 (28–42) ^b^
Marital status		
Single ^c^	9 (19%) ^d^	11 (24%) ^d^
Married	33 (79%)	34 (76%)
Education level ^e^		
School	31 (74%)	40 (89%)
Higher education	11 (26%)	5 (11%)

^a^ Three men reported 2 contacts each; ^b^ Median (interquartile range—25% to 75%); ^c^ Includes divorced; ^d^ Percent of total in each group throughout the table; ^e^ Education level was divided into two categories: school included primary school, junior/senior high school, and vocational school; higher education included undergraduate and/or graduate degrees.

**Table 2:** Chronology of exposure to men with gonococcal urethritis relative to onset of symptoms.

Exposure Relative to Male’s Symptoms	Exposed Women		Prevalence	OR	*p*-Value
	Positive ^a^	Negative ^a^	Positive ^a^	(95% CI)	
	*n* = 34 ^b^	*n* = 11 ^b^	%		
Prior	26 (76%)	8 (73%)	76	1.22 (0.26–5.72)	1.0
After	3 (9%)	2 (18%)	60	0.44 (0.63–3.02)	0.58
Prior and After	5 (15%)	1 (9%)	84	1.72 (0.18–16.59)	1.0

OR: odds Ratio; CI: Confidence Interval; ^a^ *N. gonorrhoeae* culture status; ^b^ Percent of total in each group throughout the table.

**Table 3:** Contraception methods and prevalence of *Neisseria gonorrhoeae* culture-positivity among exposed women.

Contraception ^a^	Exposed Women		Prevalence	OR	*p*-Value
	Positive ^b^	Negative ^b^	Positive ^b^	(95% CI)	
	*n* = 34	*n* = 11	%		
None	13 (38%) ^c^	4 (36%) ^c^	76	1.23 (0.3–4.99)	1.0
Condom	6 (18%)	2 (18%)	75	0.96 (0.165–5.65)	1.0
IUD	10 (30%)	5 (45%)	67	0.5 (0.12–2.0)	0.46
Hormone-based					
Pill	3 (7%)	0	100	UN	0.57
Emergency	1 (3%)	0	100	UN	1.0
Unknown	1 (3%)	0	100	UN	1.0

OR: odds Ratio; CI: Confidence Interval; UN: undefined; ^a^ Within 6 months prior to enrollment; ^b^ *N. gonorrhoeae* culture status; ^c^ Percent of total in each group throughout the table.

**Table 4:** Self-reported and diagnosed clinical symptoms, inflammation, and prevalence of *Neisseria gonorrhoeae* culture-positivity among exposed women.

Symptoms and Signs	Exposed Women		Prevalence	OR	*p*-Value
	Positive ^a^	Negative ^a^	Positive ^a^	(95% CI)	
	*n* = 34	*n* = 11	%		
**Self-reported Signs and Symptoms ^b^**	**23 (68%)**	**6 (55%)**	**79%**		
Vaginal discharge	18 (53%) ^c^	5 (45%) ^c^	78	1.28 (0.32–5.01)	1.0
Vaginal bleeding (other than menses)	1 (3%)	1 (9%)	50	0.63 (0.05–7.64)	1.0
Pain					
Abdominal	3 (9%)	0	100	UN	0.56
Urination	6 (18%)	2 (18%)	75	0.96 (0.16–5.65)	1.0
Intercourse	1 (3%)	1 (9%)	50	0.63 (0.05–7.64)	1.0
Sore throat	2 (6%)	0	100		
**Clinical Signs ^d^**	**30 (88%)**	**10 (91%)**	**75%**		
Vaginal discharge	30 (88%)	9 (82%)	76	1.67 (0.26–10.64)	0.62
Purulent	16	4	80	1.56 (0.38–6.31)	0.73
Clear-mucoid	6	4	60	0.38 (0.08–1.7)	0.23
Grey-white	7	1	86	2.59 (0.28–23.8)	0.66
Curdy	1	0	100	UN	1.0
Cervical discharge	27 (79%)	9 (82%)	74	0.86 (0.15–4.90)	1.0
Purulent	20	6	77	1.19 (0.3–4.68)	1.0
Bloody	3	0	100	UN	0.57
Clear-mucoid	2	2	50	0.28 (0.03–2.29)	0.25
White	2	1	67	0.63 (0.05–7.64)	1.0
Cervical inflammation	23 (68%)	8 (73%)	74	0.78 (0.17–3.55)	1.0
Moderate	8	1	89	3.07 (0.33–27.86)	0.42
Minimal	15	7	68	0.45 (0.11–1.83)	0.31
PMN score	**34 (100%)**	**10 (91%)**			
None [0 cells]	0	1 (9%)	0	UN	0.24
Low [1–4 cells]	2 (6%)	2 (18%)	50	0.28 (0.03–2.29)	0.25
Moderate [5–9 cells]	10 (30%)	3 (27%)	77	1.62 (0.29–8.97)	0.70
High [≥10 cells]	22 (65%)	5 (45%)	81	1.74 (0.43–6.98)	0.48

OR: odds Ratio; CI: Confidence Interval; UN: undefined; ^a^ *N. gonorrhoeae* positive or negative culture; ^b^ Self-reported by the subject; ^c^ Percent of total in each group throughout the table; ^d^ Observed on clinical exam.

**Table 5:** Co-infections diagnosed in exposed women and prevalence of *Neisseria gonorrhoeae* culture-positivity among exposed women.

STI ^b^	Exposed Women		Prevalence	OR	*p*-Value
	Positive ^a^	Negative ^a^	Positive ^a^	(95% CI)	
	*n* = 34	*n* = 11	%		
*C. trachomatis*	11 (33%) ^c^	4 (36%) ^c^	73	0.84 (0.2–3.47)	1.0
*M. genitalium*	6 (18%)	1 (9%)	86	UN	1.0
*T. vaginalis*	2 (6%)	2 (18%)	50	UN	1.0
*U. urealyticum*	24 (71%)	4 (36%)	86	5.45 (0.62–47. 9)	0.14
*C. albicans*	2 (6%)	0	100	UN	1.0
Bacterial vaginosis	13 (38%)	6 (55%)	68	0.35 (0.07–1.74)	0.24

OR: odds Ratio; CI: Confidence Interval; UN: undefined^; a^ *N. gonorrhoeae* culture status; ^b^ Diagnosed by culture or PCR; ^c^ Percent of total in each group throughout the table.

## Data Availability

Data available upon request.

## Supplementary Materials

The following are available online at https://www.mdpi.com/article/10.3390/vaccines9040327/s1, Table S1: Clinical exam evaluated for exposed woman during her visit to the clinic.
