# Supplementary material for: Epidemiological and Clinical Observations of Gonococcal Infections in Women and Prevention Strategies
Source: Vaccines (Basel). 2021 Apr 1;9(4):327. doi: 10.3390/vaccines9040327 (PMC8066387; doi:10.3390/vaccines9040327)
Supplement: Supplementary file 1 [file vaccines-09-00327-s001.pdf]

Table S1: Clinical exam evaluated for exposed woman during her visit to the clinic

Clinical exam	Number of subjects and result
Pain on lateral motion of cervix:	44 absent; 1 not examined
Uterine tenderness	44 absent; 1 minimal
Adnexal tenderness [a]	45 absent
Adnexal mass [a]	44 absent; 1 not examined
Abdominal tenderness [b]	43 absent; 1 minimal; 1 moderate
Hepatic tenderness [c]	43 absent; 2 not examined
Inguinal Nodes [d]	45 absent
Genital lesions [e]	45 absent
Vaginal Discharge [f]	39 present; 6 absent;
Purulent	11 scant; 9 moderate
Clear or mucoid	6 scant; 4 moderate
Grey-white	8 scant
Curdy	1 scant
Cervical discharge	36 present; 9 absent;
Purulent	10 scant; 16 moderate
Clear or mucoid	10 scant; 16 moderate
Bloody	3 scant; 1 moderate
White	1 scant; 2 moderate
Menstrual	2 scant
Inflammation of cervix	31 present; 14 absent
Rectal tenderness	41 absent; 1 minimal; 1 moderate; 2 not examined
GNID	36 present; 9 absent
PMN [g]	1 absent; 4 low; 13 moderate; 27 high
Vaginal, Wet prep:	23 normal; 22 abnormal
Trichomonas vaginalis	4 present 41 absent
Candida albicans	2 present; 43 absent
Clue cells (indicative for BV)	19 present; 26 absent

Syphilis – dark field	9 absent; 36 not examined
Syphilis – serology	2 present; 23 not examined
HSV-1 IgG	10 absent; 35 not examined
HSV-1 IgM	10 absent; 35 not examined
HSV-2 IgG	10 absent; 35 not examined
HSV-2 IgM	10 absent; 35 not examined
HPV	10 absent; 35 not examined
Chlamydia trachomatis	16 present; 29 absent
Ureaplasma urealyticum	28 present; 17 absent
Mycoplasma genitalium	7 present; 38 absent
Mycoplasma homini	9 present; 36 absent

PMN: Cervical Gram's Stain for polymorphonuclear neutrophils; GNID: Cervical Gram's Stain for Gram negative intracellular diplococci; BV: bacterial vaginosis

[a] Right / Left

[b] Right / Left / Middle Upper Quadrant or Lower Quadrant

[c] Palpation / Percussion

[d] Right / Left / Skene's Gland abscess / Bartholin's cyst or abscess

[e] Chancres (syphylis) / Condyloma lata (syphylis) / Condyloma acuminata (HPV warts) / Vesicles (Herpes) / Ulcers (etiology unclear)

[f] Bloody, Foamy and Menstrual absent in all subjects

[g] Low: presence of 1-4 PMN; Moderate: presence of 5-9 PMN; High: presence of ≥10 PMN
